# Evolution of neurometabolic frugality in harvester ants

**DOI:** 10.1098/rspb.2026.0387

**Published:** 2026-10-21

**Authors:** Craig D. Perl, Zach N. Coto, Robert Johnson, Meredith Johnson, Leland Graber, James Haas, Jane S. Waters, James Traniello, Jon F. Harrison

**Affiliations:** ^1^ School of Life Sciences, Arizona State University, Tempe, AZ, USA; ^2^ Biology, Boston University, Boston, MA, USA; ^3^ Arizona State University, Tempe, AZ, USA; ^4^ Cornell University, Ithaca, NY, USA; ^5^ Biology, Providence College, Providence, RI, USA

**Keywords:** brain evolution, brain metabolism, social complexity, metabolic rate, brain size, social evolution

## Abstract

Despite the importance of neurometabolic cost in brain size evolution, quantitative data on brain metabolic rates (MRs) are lacking. We measured *ex vivo* brain MRs among species of the ant genus *Pogonomyrmex* to test three hypotheses: (i) more socially complex species have higher brain MRs; (ii) smaller-bodied species have higher brain mass-specific metabolic rates (MSMRs); and (iii) more recently derived species have evolved more metabolically ‘frugal’ brains. Worker body size and colony size, a proxy for social complexity vary significantly among *Pogonomyrmex* species and were positively correlated. Species with larger colonies did not have larger brains or higher brain MRs. Species with smaller workers had relatively larger brains and higher brain MSMR: 38% of resting MR was attributable to brain metabolism in species with the smallest workers, compared to 6% in species with the largest workers. We suggest that smaller worker body sizes may require higher brain mass-specific energetic costs to achieve comparable performance by absolutely smaller brains. More derived species had strikingly lower mass-specific brain metabolic costs, suggesting that increases in worker body size have selected for neurometabolic frugality through reductions in brain MSMR.

## 1. Introduction

Understanding the selective forces that drive brain evolution and its macroevolutionary patterns remains one of the most intriguing unresolved problems in biology [1–3]. Body size strongly affects brain size; relative brain size decreases with body size across a large range of taxa (Haller's rule) [4,5] but the mechanisms linking this size-scaling pattern to brain size, morphology and physiology remain unclear [3,5,6]. There are multiple hypotheses proposing that social and ecological pressures exert a strong influence on brain evolution. Theories, such as the social brain hypothesis, predict that higher behavioural and cognitive demands of group living [7,8] select for increased brain size and/or metabolism, while other hypotheses highlight the importance of higher-energy diets (or diets that require more sophisticated foraging) as drivers of brain evolution [9]. Collaterally, the expensive tissue hypothesis [10] emphasizes the unusually high metabolic expense of the brain and posits that trade-offs between costly neural and digestive tissue permitted the evolution of larger brain sizes. Additionally, an adipose-brain trade-off may also explain brain size evolution with fat stores and encephalization forming alternative insurance policies against starvation [11]. Support for these theories varies and debate continues [11–13], most often focusing on vertebrates [1,14], particularly humans and other primates [15] (and references therein). While energy costs are clearly important components of many theories of brain evolution, few studies have measured brain metabolic rates (MRs). Furthermore, no studies have examined directional evolution of the size and MR of brains, nor have they examined their interaction with body size or social evolution.

There is considerable evidence that larger animals have absolutely larger but relatively smaller brains, and some evidence that brains of larger animals use less energy per gram. Brain size scales hypometrically in mammals [4,16], birds [17], reptiles [18], amphibians [19], fish [20], arachnids [21] and insects [22], including bees [23–25] and ants [26,27]. Brain MR has also shown to scale hypometrically in mammals and insects [6,28]. The causes of the hypometric scaling of brain size and metabolism are hypothesized to relate to constraints on whole-body MR [4] and/or selection on life history parameters, such as the need for smaller animals to maintain a behavioural capacity comparable with that of larger animals [29–31]. There may also be selection for energy efficiency and a longer lifespan in larger animals [29,31,32]. Larger brains may be more cognitively capable [33–35], but body and brain size effects on behavioural capacities are inconsistent. Generally, small-bodied species do not have limited behavioural repertoires compared to larger-bodied species [29,36], and the selection on smaller individuals and species to maintain behavioural and cognitive performance for competition with larger competitors [31] may explain hypometric brain scaling. The brains of the smallest insects displace somata and lose nuclei to preserve axon number, increasing relative brain mass allocated to energetically costly neurons, which may generate higher brain mass-specific metabolic rates (MSMRs; the MR of a tissue divided by the mass of said tissue) in smaller individuals [37–40]. Because brains can cost as much as 20% of the resting MR of an animal [41], it is plausible that there should be selection over time for efficiency in brain function, leading to a reduction in brain MSMR. However, no study has examined the scaling of brain MR in a phylogenetic context; therefore, there are no data on the extent to which variation in brain metabolism is associated with relatedness independent of body and brain size variation or whether there are directional evolutionary tendencies in neurometabolism.

Eusocial insects have emerged as important models for analyses of brain evolution in animal societies owing to their exceptional social diversity and body-size variation [22,27,42–45]. The application of the social brain hypothesis to eusocial insects is controversial [45–47]. Worker sterility within eusocial species precludes the evolution of individual strategies of reproductive competition, which can require coalition formation, high socio-cognitive skills and increased processing capability. Eusocial insect workers and groups exhibit individual and collective cognition, respectively [48], but worker cognitive abilities do not usually directly enhance worker fitness, but rather colony reproductive success. In eusocial species with larger colony sizes, a proxy for social complexity owing to increased social interactions [26,43,49–52], individual behavioural/cognitive load may decrease owing to task specialization and division of labour [51]. This could result in smaller, less energy-intensive brains associated with increasing social complexity [42]. Here, empirical data are equivocal [15,45]. Comparisons of the evolutionary neurobiology of ants suggest that striking differences in colony size and degree of social complexity influence brain size and its energetic expense [53]. In some ants, species that form larger colonies have evolved workers with relatively larger brains [26]. Sociality is also associated with larger brains in some bees [24,25,54,55], but the broader pattern across bees also indicates a significant role for diet and life history [23,56]. Conversely, in monomorphic fungus-growing ants [57] and vespid wasps [43], increased social complexity is associated, respectively, with decreased relative size of brains and mushroom bodies, the brain compartments specialized for learning and memory [56]. Together, these results suggest that the effects of sociality on social insect brains are complex and probably affected by ecology, colony size, social complexity and clade [44,46].

Phylogenetic history also plays an important role in brain evolution. Different clades often vary in absolute and relative brain sizes [3,5,23,27] and can form different relationships between brain size and body size [3]. The Marsh–Lartet rule, which suggests an evolutionary trend of increasing relative brain mass in mammals, was recently supported for three mammalian orders, including primates [3,58]. In bees, phylogenetic analysis revealed that brain size evolution is linked to evolutionary changes in voltinism and host specialization [23]. Understanding the directional evolution of brain size and MR, as well as understanding how tightly these are constrained by body size, are important questions for evolutionary biology. Unfortunately, we currently lack studies of directional evolution and phylogenetic patterns for brain metabolism in any animal clade.

To address major gaps in our understanding of the evolution of neurometabolism, brain size and social systems, we measured brain masses, whole-body MRs and intact brain MRs *ex vivo* from workers of eleven species of seed-harvesting ants, *Pogonomyrmex* [59]. Ants in general [60] provide excellent models to address questions at the intersection of neurobiology, socioecology and metabolism owing to their large number of species, fantastic ecological variation, sociality and relatively complex behaviours. Larger body size is known to be associated with a relatively smaller brain within leafcutter ant sub-castes [27], within *Cataglyphis* species [26], and across 70 species of ants [27], a pattern reflected more generally in insects [5]. We selected species of *Pogonomyrmex* for our study because of their significant variation in colony size and body size, a range comparable with that exhibited within mammalian orders (supplementary material, table S1). Critically, the species we studied are all primarily granivorous and reproduce annually, enabling tests for effects of body size, colony size and phylogeny without obvious confounding variables of life history or diet (supplementary material, table S1). All species sampled for our investigation inhabit grasslands of the southwestern USA and robust phylogenies of the genus now exist [61].

Using 11 species of *Pogonomyrmex*, we test three hypotheses concerning brain evolution: (i) high social complexity requires high brain MRs to meet increased cognitive demands, (ii) smaller individuals require higher brain capacities to compete with larger species owing to their absolutely smaller brains, and (iii) over time, the high energetic cost of brains should select for frugality with brains of derived species selected to have lower metabolic costs. Hypothesis 1 predicts that species with larger colony size should have larger brains or brains with higher metabolic capacities. Hypothesis 2 predicts that species with smaller workers should have relatively larger brains and higher brain MSMRs. Hypothesis 3 predicts that more derived species should have smaller brains and lower brain MSMRs.

## 2. Results

### (a) Phylogenetic signal and directional evolution

To assess hypothesis 3 and because relatedness must be accounted for in statistical analyses if it influences parameters of interest, we first assessed the importance of phylogeny in *Pogonomyrmex* brain evolution. There was a significant phylogenetic signal for brain MSMR (figure 1; Pagel's *λ* = 1.00, *p* < 0.01), body MSMR (figure 1; Pagel's *λ* = 1.00, *p* < 0.01), brain MR (figure 1; Pagel's *λ* = 0.98, *p* < 0.01) and colony size (figure 1; Pagel's λ = 1.00, p < 0.01). For brain MSMR and body MSMR, as predicted by hypothesis 3, there was a trend for more derived species to have lower rates (figure 1). While we did not have predictions for hypothesis 3 for the evolutionary direction of absolute brain MR or colony size, for both variables there was a trend for more derived species to have higher values, suggesting evolution toward greater social complexity (figure 1). There was no significant phylogenetic signal for body mass (Pagel's *λ* = 0.55, *p* > 0.20), brain mass (Pagel's *λ* = 0.65, *p* > 0.20) or whole-body MR (Pagel's *λ* < 0.01, *p* = 1.00). As such, subsequent analyses using body mass, whole-body MR or brain mass were conducted without any phylogenetic correction, but any analysis of brain MR, brain MSMR, body MSMR or colony size accounted for phylogeny.

**Figure 1.**
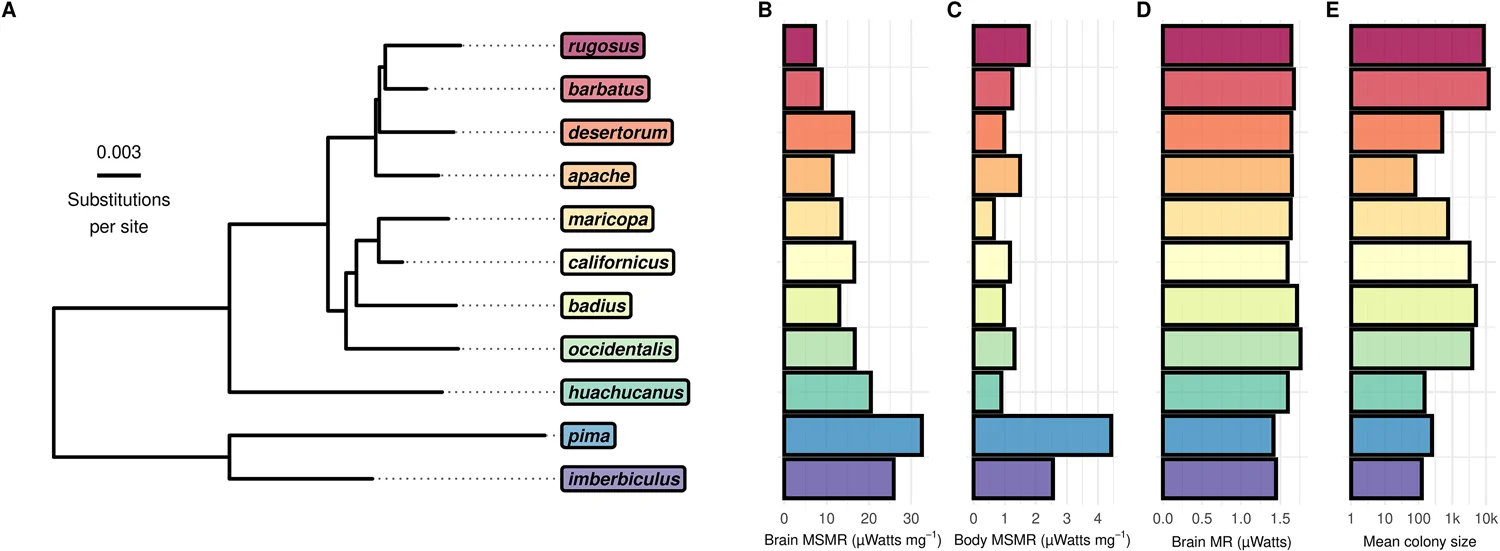
(A) Brain mass-specific and body MSMRs were strongly affected by phylogeny. (B,C) The basal clades of the genus had relatively high brain and body MSMRs, while the *rugosus*–*barbatus* clade had the lowest, suggestive of a progressive evolutionary trend toward lower brain and body MSMRs. (D) Absolute brain MRs also had a significant phylogenetic signal, being generally lower in more basal species. (E) Mean colony sizes had a significant phylogenetic signal being generally smaller in more basal species. Colours are not indicative of any value but included to aid readability. These are the only variables that presented a significant phylogenetic signal.

### (b) Worker mass, brain size scaling and relationship with colony size

As predicted by hypothesis 2, smaller workers had relatively larger brains. There was a significant relationship between log_10_ brain mass and log_10_ body mass; brain mass increased with body mass (figure 2A; linear mixed effects model; *t*_9,252_ = 15.73, *p* < 0.001, Benjamini–Holm adjusted *p* < 0.001). However, brain mass scaled strongly hypometrically, with an allometric slope of 0.54 ± 0.03. For this and subsequent regressions, the standard error is provided.

**Figure 2.**
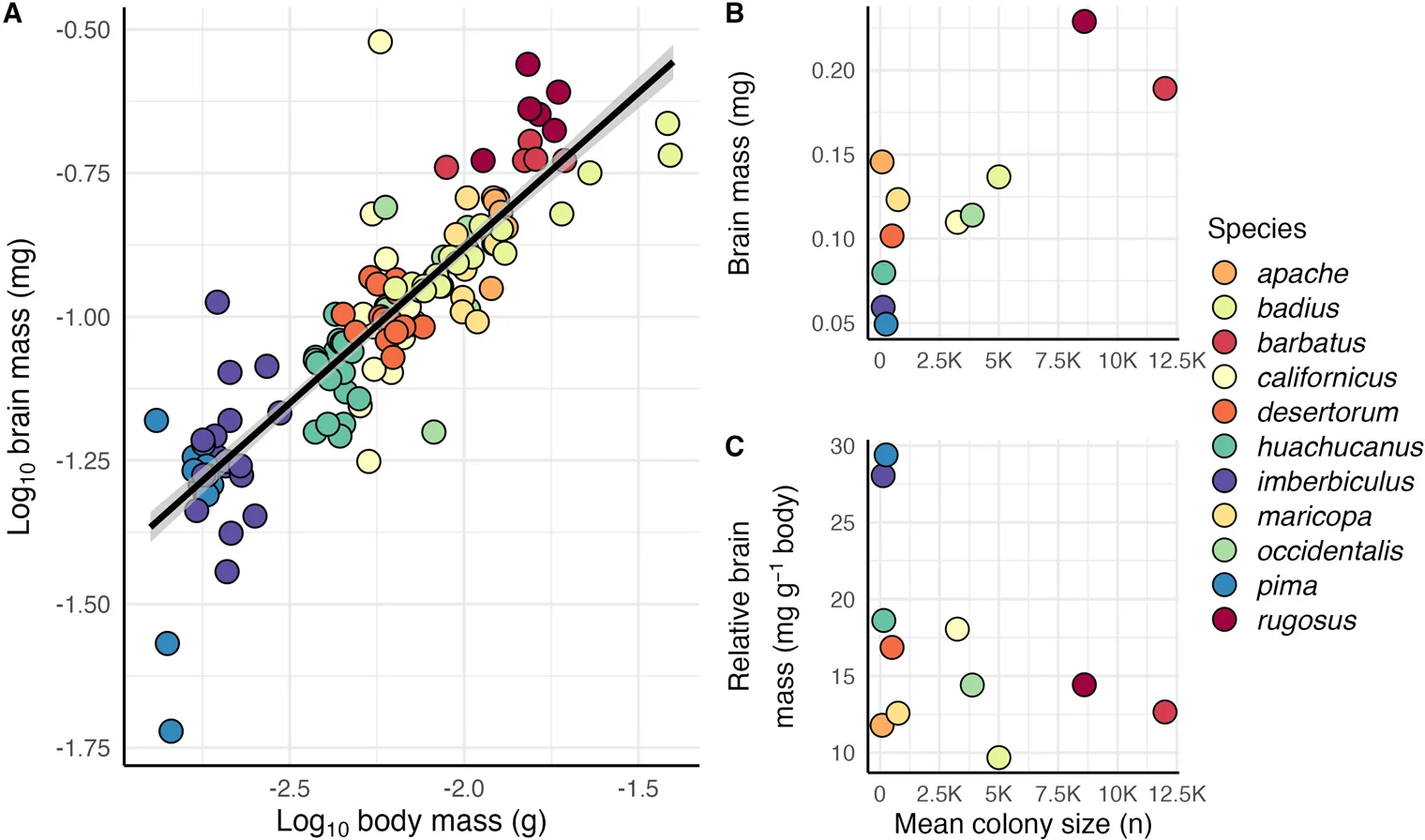
(A) Brain mass scaled hypometrically with worker body mass (log_10_ brain mass = 0.54 × log_10_ body mass + 0.20) and (B) brain mass increased with increasing colonial population (brain mass = 0.00000666 × colony size + 0.08179035). (C) Relative brain mass was not significantly related to colonial population size.

As predicted by hypothesis 1, there was a significant positive relationship between brain mass and colony size (figure 2B; phylogenetic generalized least squares (PGLS); *t*_9,11_ = 3.07, *p* < 0.02, Benjamini–Holm adjusted *p* < 0.03); worker brain mass increased with increasing colony size. However, this was owing to an increase in mean worker mass with increasing colony size (PGLS; *t*_9,11_ = 2.73, *p* < 0.03; supplementary material, figure S1). There was no significant relationship between relative brain mass (brain mass per unit body mass) and colony size (figure 2C; PGLS; *t*_9,11_ = 0.52, *p* = 0.62), providing evidence against hypothesis 1.

### (c) Whole body resting metabolic scaling and relationship with colony size

We did not make specific predictions concerning body-size effects on whole-body MR, but given the high energetic cost of brains, hypothesis 2 could plausibly predict lower whole-body MSMRs in species with larger worker body size. There was a significant relationship between log_10_ whole-body MR and log_10_ body mass (figure 3A; linear mixed effects model; *t*_37,252_ = 3.10, *p* < 0.01, Benjamini–Holm adjusted *p* < 0.02); body MR increased with increasing body mass. The scaling of whole-body MR was even more hypometric (relatively low in larger animals) than commonly observed with an allometric slope for whole-body resting MR of 0.35 ± 0.11.

**Figure 3.**
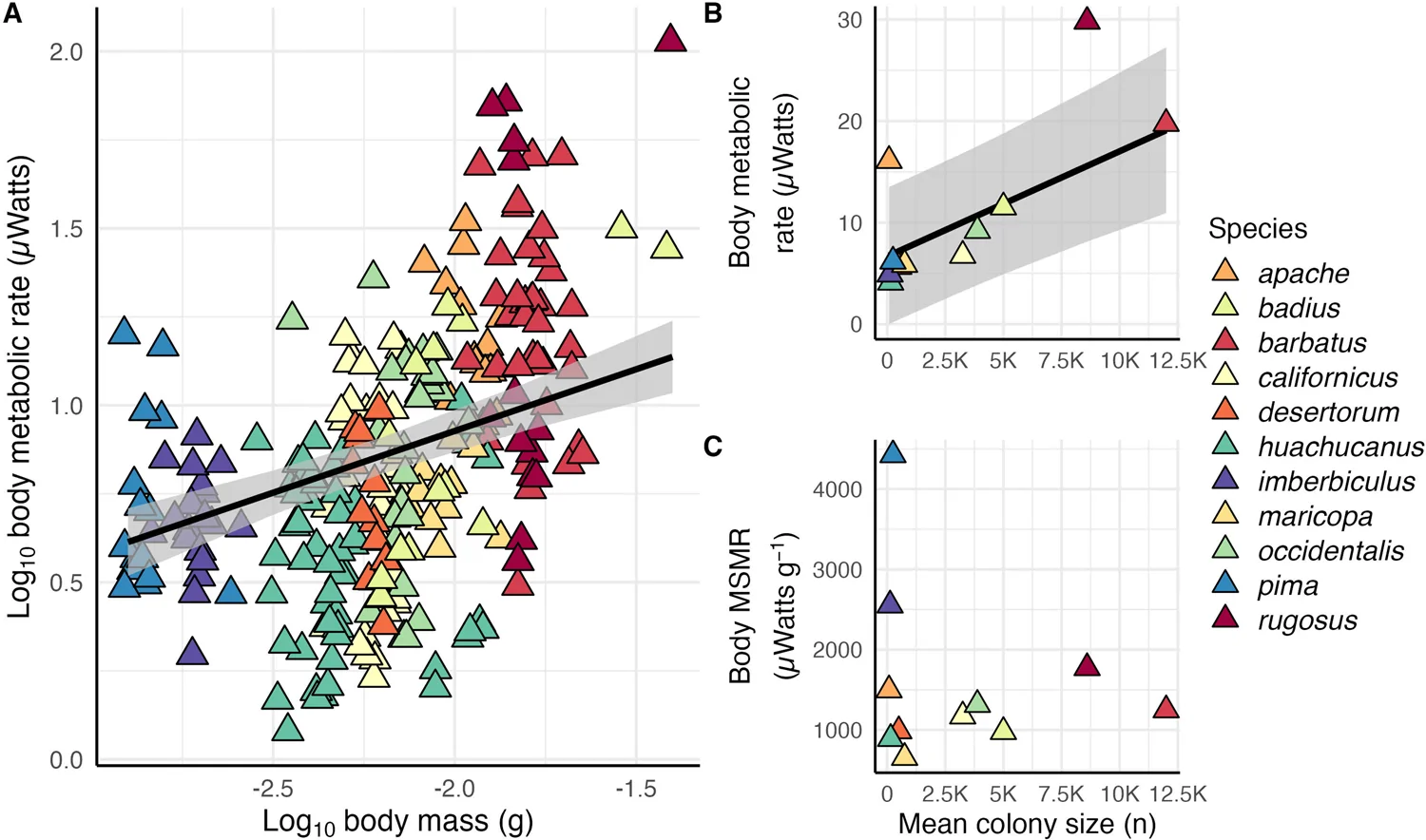
(A) Whole-body MR increased in larger workers but scaled hypometrically (log_10_ body MR = 0.35 × log_10_ body mass + 1.62). (B) Whole-body MR increased with increasing colonial population (body MR = 0.001035 × colony size + 6.67), though this relationship is non-significant when examined using a multivariate approach that accounts for body size and when *p*-value adjustments for multiple comparisons are applied. (C) Whole-body mass-specific MR was not significantly related to colonial population size.

Again, we did not make a specific prediction about the association of colony size and whole-body MR. Given the quantitative importance of brain MR to whole-body MR, it would be plausible to predict higher whole-body MSMRs in ants from larger colonies. There was a borderline significant relationship between whole-body MR and colony size (figure 3B; PGLS; *t*_9,11_ = 2.44, *p* < 0.04, Benjamini–Holm adjusted *p* = 0.06); body MR increased with increasing colony size. However, after accounting for body size, the relationship between whole-body MR and colony size becomes non-significant, a conclusion reflected in the adjusted *p*-value from the univariate model. When colony size and body mass were both included as predictors of body MR in a multivariate model, body mass emerged as a significant factor (PGLS; *t*_1,11_ = 4.09, *p* < 0.01) whereas colony size did not (PGLS; *t*_1,11_ = 0.95, *p* = 0.37). There was no significant relationship between whole-body MSMR and colony size (figure 4C; PGLS; *t*_9,11_ = 0.35, *p* = 0.73). Furthermore, there was no significant relationship between body-mass-corrected MR (the residuals of whole-body MR regressed against body mass) and colony size (PGLS; *F*_1,11_ = 1.13, *p* = 0.29; supplementary material, figure S2A). Together, these data provide some evidence against hypothesis 1 and for hypothesis 2.

**Figure 4.**
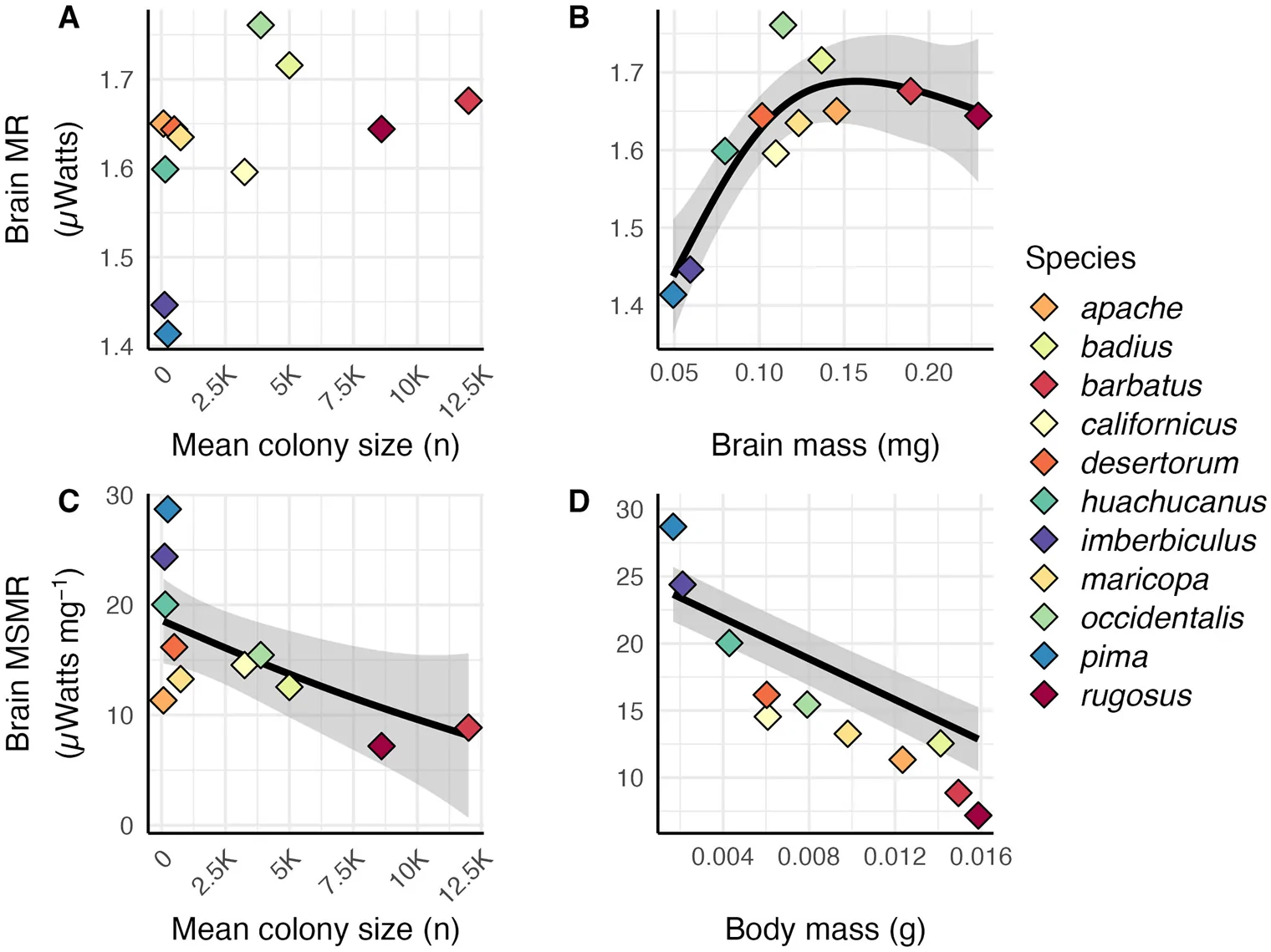
PGLS analyses of independent effects on brain metabolism. (A) There was no relationship between brain MR and colonial population. (B) There is a significant relationship between brain MR and brain mass. (C) Brain MSMR decreased with increasing colony size. (D) Brain MSMR also declined with increasing worker body mass (brain MSMR = –874.87 × body mass + 26.94). When analysing body mass and colony size as predictors of brain MSMR in a multivariate model, colony size ceases to be a significant predictor of brain MSMR and body mass remains a significant factor.

### (d) Brain metabolic rate scaling and relationship with colony size

Providing strong evidence against hypothesis 1, there was no significant relationship between brain MR and colony size (figure 4A; PGLS; *t*_9,11_ = 0.77, *p* = 0.46). Brain MSMR decreased linearly with colony size (figure 4C, PGLS; *t*_9,11_ = 2.33, *p* < 0.05), but a nonlinear model provided a better fit (Akaike information criterion PGLS = 75.02 and Akaike information criterion generalized additive model = 70.89) and is presented here (generalized additive models with thin plate spline and phylogenetic penalty; *F*_1_,_11_ = 7.06, *p* < 0.03, Benjamini–Holm adjusted *p* < 0.04).

Species with larger workers had higher absolute brain MRs and as predicted by hypothesis 2, lower brain MSMRs. There was no significant linear relationship between brain MR and brain mass (PGLS; *t*_9,11_ = 0.84, *p* = 0.42), but there was a significant nonlinear relationship between brain MR and brain mass (figure 4B; generalized additive model with thin plate spline and phylogenetic penalty; *F*_1_,_11_ = 3.21, *p* < 0.01, Benjamini–Holm adjusted *p* < 0.01). Brain MR increased with increasing brain mass up to a value of 0.148 mg and then decreased slightly. There was also a highly significant relationship between brain MSMR and body mass (figure 4D; PGLS; *t*_1,11_ = 5.70, *p* < 0.001, Benjamini–Holm adjusted *p* < 0.002). As body mass increased, the relative energetic cost per gram of brain tissue decreased. Brains from smaller workers used more energy per unit mass of brain tissue than those from larger individuals.

In support of hypothesis 2 and against hypothesis 1, when colony size and body mass were both included as predictors of brain MSMR in a multivariate model, body mass emerged as a significant factor (PGLS; *t*_1,11_ = 5.70, *p* < 0.001) whereas colony size did not (PGLS; *t*_1,11_ = 0.68, *p* = 0.52). There was no significant interaction term (PGLS; *t*_1,11_ = 0.90, *p* = 0.40). Furthermore, there was no significant relationship between body-mass-corrected brain MSMR (residuals of brain MSMR regressed against body mass) and colony size (PGLS; *F*_1,11_ = 0.33, *p* = 0.58; supplementary material, figure S2B).

## 3. Discussion

Species of the ant genus *Pogonomyrmex* vary in social phenotype and worker body size. We provide the first evidence in support of neurofrugality in brain evolution (hypothesis 3), and the first evidence in insects that smaller-bodied species require relatively higher brain capacities to enable behavioural performance comparable with that of larger-bodied conspecifics (hypothesis 2). We also provide evidence against hypothesis 1, which stated that more socially complex species have greater brain capacities. Phylogeny and body size had strong effects on brain and body energetics, but colony size, a proxy for social complexity, did not. In support of hypothesis 3, there was a general trend for more derived species to have lower brain and body MSMRs (figure 1). In support of hypothesis 2, smaller-bodied species had relatively larger brains and higher MSMRs (figure 4D). Workers in species characterized by larger colony size had lower brain MSMRs (figure 4C). As described, it is clear that many factors affect brain evolution, but our studies provide a strong test of these three hypotheses while controlling for phylogeny, diet, ecology and life history.

Colony size was strongly correlated with body size; larger colonies had larger workers (figure 1). There were some significant relationships between colony size and energetics: workers from larger colonies showed higher body MRs. However, this relationship was not independent of an increase in worker body size (supplementary material, figure S2A), and colony size was not a significant predictor of body MR with body mass accounted for. Therefore, body size was the more important factor for determining body and brain energetics.

Body size is the most important factor for determining energetics, but how this trait affects the association of larger workers with larger colonies remains unclear. Worker size is inconsistently associated with colony size in social insects; some ant species (e.g. *Solenopsis invicta* and *Linepithema humile*) with the most populous colonies have relatively small workers, and others are exceptionally polymorphic. Kaspari [62] found no significant relationship between worker number and mass in a study of 434 New World ant species, indicating that larger workers are not always associated with larger colonies. In a study of more than 100 neotropical ant species, worker mass, queen mass and colony mass were found to be strongly positively correlated [63]. To our knowledge, our study is the first to test for associations between worker size and colony size while controlling for phylogeny and to a substantial degree, for diet, ecology and life history.

The relationship between worker body size, foraging productivity and energy efficiency is important but unclear and may not reflect vertebrate patterns owing to the impacts of larger colony sizes on worker resource acquisition. Across multiple vertebrate taxa, species with larger body sizes use more resources per individual, but as individual abundances and generation times scale inversely to individual size in vertebrates, large- and small-bodied species tend to use similar amounts of energy per year (the equal fitness paradigm [64,65]). There appear to be advantages to larger colony sizes. Larger colonies, associated with larger body sizes in *Pogonomyrmex* may control clumped resources [66,67] (but see [68]) and avoid conflict with neighbouring colonies [66], but it is unknown how resource acquisition compares across small and large colonies and whether they also conform to the equal fitness paradigm. Within *Pogonomyrmex,* group or trunk-trail foraging is restricted to larger-bodied species and larger colonies [69]; therefore, it is possible that the energetic benefits brought by the evolution of larger workers with access to differential foraging strategies can support larger colony sizes. However, in other seed-harvesting species, smaller individuals are associated with group-foraging strategies [69]. It is therefore more likely that group foraging allows for resource domination in a typically stochastic environment. Individual foraging trips, irrespective of foraging strategy, have minimal impact on worker energy budgets [70,71] but group-foraging strategies can ease the exploitation of abundant resources when they appear [69]. *Pogonomyrmex* colonies can experience significant interspecific food resource competition [67], thus influencing the evolution of worker size through character displacement, reducing competition by niche segregation or selecting for differential foraging strategies [72]. Colonies of *P. barbatus*, a species with the largest workers, have lower fitness when neighbouring colony density is higher [73]. Resource competition may favour the evolution of traits, such as territoriality and trunk-trail foraging, enabling resource control and, collaterally, larger workers and colony sizes. Comparative data on foraging success, colony fitness, generation time, abundance and fitness of these species in the field are lacking, and the hypothesis that large worker size, social complexity and ecological dominance are linked in *Pogonomyrmex* requires additional research, especially to determine the causality among the evolution of colony size, body size, foraging strategy and their metabolic consequences.

We did not find a significant effect of colony size on whole-body MSMR, perhaps suggesting that larger colonies are not more metabolically efficient in our sample of *Pogonomyrmex* species. However, it is important to note that colony MRs cannot be predicted from the resting MRs of individual workers [74]. Large colony sizes in social insects correlate with lower colony-level MSMRs [75] as well as increased division of labour in *P. californicus* [51,76]. Worker task diversity increases as colonies grow [76] with older, larger colonies having workers that tend to specialize in specific tasks, rather than engaging in a wider range of tasks [51,74], in addition to having lower activity rates [74,77]. Such behavioural specializations could allow larger colonies to reduce mass-specific energy use. There were extremely high effect sizes of phylogeny on brain MR, brain MSMR and whole-body MSMR. We did not find phylogenetic signal for brain mass, diverging from evolutionary trends in mammals, where directional evolution for increasing relative brain mass has been reported within some mammalian orders, including primates [3,58].

Although our analyses were performed on a single genus, the body size range across the *Pogonomyrmex* species sampled in our study is comparable to variation exhibited within mammalian orders, allowing comparisons of scaling across these diverse clades. The distinct life histories and reproductive traits of mammals and eusocial insects may explain the differential neurometabolic response to increasing social complexity. Our data indicate that the social brain hypothesis may have limitations when applied to the brain energetics of eusocial insects [42]. We find concordance with other investigations into the association of sociality and neurometabolism in ants. Kamhi *et al.* [53] report that Australasian weaver ants, *Oecophylla smaragdina,* an exemplar of insect social complexity, have larger brains and greater investment in the mushroom bodies but significantly lower cytochrome oxidase (COX) activity in the mushroom body medial and lateral calyces relative to those of the socially simpler, small-colony sister species *Formica subsericea*. The mushroom bodies are a brain compartment dedicated to higher-order processing, integration, learning and memory and thus probably provide neural support for complex social behaviour in ants [53]. COX is a proxy for ATP usage, therefore mass-specific brain energy expenditure may decrease as social complexity increases. Our data support this pattern by *directly* measuring brain energetics. We find the same relationship in *Pogonomyrmex:* brain MSMR decreases with increasing social complexity, rather than increasing as would be expected from social brain theory.

Phylogeny is important for both brain and whole-body MSMR, indicative of selection towards energetic frugality; the highest values occurred in the most basal clade (*P. imberbiculus* and *P. pima*), followed by the next most basal species (*P. huachucanus*). The lowest values occurred in the most derived clades, strongly suggesting an evolutionary trend toward lower brain and whole-body MSMRs. Absolute brain MR showed the opposite pattern: the lowest values were found in the more basal clades. Interestingly, brain and body mass showed no phylogenetic signal, suggesting that the above patterns are not simply driven by brain and body mass and that mass is more evolutionarily labile than MR. Phylogenetic signal for body mass-related traits and MRs has been found to be minimal in some insects [78,79], but strong in others [78]. Our sampling of 11 *Pogonomyrmex* species should be extended to confirm these patterns, as we measured approximately a third of the known North American species in this genus [80].

Larger-bodied ant workers have remarkably lower energy usage per unit mass of brain. The smallest-bodied species have 10 times higher brain MSMR than the largest-bodied species in *Pogonomyrmex*. Owing to their relatively large brains and higher brain MSMRs, brains account for a greater proportion of the metabolic budget in smaller-bodied species (38% in *P. huachuncanus* and 29% in *P. desertorum* and *P. imberbiculus*) but only 6% in the largest-bodied species (*P. rugosus*). Even though brains account for only 2% of whole-body mass, this scaling of brain MR explains approximately 25% of the hypometric scaling of whole-body MRs across the clade. This further demonstrates that body size is of high significance for determining neurometabolic costs and indicates that a small body size is associated with both relatively larger and more energetically active brains. The high energetic cost incurred by brains may provide a selective pressure to reduce relative brain MRs, as observed in the more derived *Pogonomyrmex* species.

The strong hypometric scaling of brain MR suggests either selection to reduce brain MRs in larger-bodied species, selection for high brain MRs in smaller-bodied species or both. Limits to whole-body MR, such as increasingly constrained oxygen or nutrient delivery in larger brains, are a possible cause of hypometric brain MR scaling [4]. However, we currently lack data on the scaling of oxygen and substrate supply in ants as has been performed for some other groups of insects [81,82], and no studies have yet addressed this issue for any brains. Brains, and neural tissue in general, are often cited as being a significant constituent of overall energy budgets [6,10]. As such, large-bodied species with the absolute largest brain masses might gain the most benefit from reducing brain MSMRs [6,83]. Larger-bodied species generally have longer lifespans (reviewed by [32]) and there is evidence that this is also true in ants [84]. Higher MSMRs are often associated with higher rates of reactive oxygen species production, which can damage tissues and shorten lifespans, therefore it is possible that the lower MSMRs of larger-bodied species arise ultimately from selection to reduce metabolism-linked tissue damage and extend lifespan [85].

Alternatively, or additionally, species with smaller workers may experience greater selection to *increase* their brain MRs. Small harvester ant workers carry out the same tasks as larger-bodied species (e.g. forage for seeds, defend territory and/or food sources, navigate, build and maintain the nest and care for the queen and brood) with brains and sensory organs that are absolutely smaller, perhaps demanding increased relative information processing or action potential generation. Furthermore, smaller-bodied seed-harvesting species are often sympatric with larger-bodied species and probably compete to some extent for seeds [67]. Though seed sizes are positively correlated with worker body size [72], interspecific foraging competition [67] could lead to stronger selection on mass-specific brain performance in smaller-bodied species [31].

The nonlinear relationship between brain MR and brain size provides further evidence that species with smaller-brained workers may be under significant selection to maximize their performance, and therefore brain MRs did not vary between the largest species as much as between the smallest species. The steep increase in brain MR with brain size when brain mass < 0.15 mg indicates that there is a benefit to increasing brain MR at smaller brain sizes or brain energy consumption is limited at larger brain sizes.

The high brain MSMR of smaller-bodied workers may also be a function of other aspects of miniaturization [37]. Smaller insects preserve axons over glial cells in the central nervous system, probably to maintain brain function. Generating action potentials and synaptic signalling are the costliest neuronal functions [37–40]. Therefore, the necessity to maintain brain function despite miniaturization might result in brains with a relatively higher proportion of tissue engaging in high-energy activities, which causes an increase in brain MSMR. Higher brain MSMRs may also reflect the greater proportion of the brain devoted to information processing in miniaturized systems. Coupled with the strong propensity for sensory organs to also scale hypometrically [86–88], higher mass-specific information processing for smaller animals could partially preserve cognitive and behavioural capacities. This may be a proximate mechanism by which smaller individuals maintain cognitive capacity to compete with larger-bodied sympatric species [31]. Additionally, smaller-bodied species are often more likely to experience predation, which may also select for better sensory systems and reaction times [32]. Further behavioural, ecological and physiological studies will be required to determine the causal factors driving body-size associated energetic patterns.

Although our metabolic measurements were made on intact undamaged brains, we recognize that the dissection necessary for recording *ex vivo* brain MRs has potential physiological effects that are not well understood. Severing sensory and motor neurons of the brain will probably first increase ion fluxes and therefore cause apoptosis, though neurons have mechanisms to protect against this process [89]. The number of neurons cut, however, is probably extremely small relative to the total number of brain cells [89]. Furthermore, the stability of brain MRs over several hours demonstrates that dissections are not driving a fast, progressive deterioration of brain function. Data from *in vitro* tissues, including the brain, in fish indicate similar metabolic scaling exponents as those measured from intact animals [90,91], and glucose uptake by the brains of starved rats is similarly depressed *in vivo* and *ex vivo* [92], suggesting that the relationship among metabolic levels is brains are not negatively impacted by removal from the body. Many studies have successfully used the Seahorse system to detect the effects of toxins [93], diet [94] and trauma, even from partial brains that have undergone destructive sampling [95]. Together, these studies suggest that such *ex vivo* brain metabolism measures accurately reflect *in vivo* interspecific patterns, opening avenues for future research.

Size is frequently used as a proxy for brain energetic cost, neuropil investment and behavioural and/or cognitive processing ability [14]. If absolute brain size is considered as the index of brain investment, one might conclude that the social brain theory was supported in this clade; however, incorporation of the effects of size and MSMR results in the opposite conclusion. Linkages between brain size (and neuron number) and cognitive ability grow weaker as greater taxonomic diversity is considered [60], indicating that brain size might be an inconsistent metric for behavioural capability. Our brain MR data provide empirical evidence that brain size is not necessarily an accurate proxy for brain energy use [14,60]. *Pogonomyrmex rugosus* workers have the largest brain in our study (0.229 mg), approximately double the mass of *P. occidentalis* (0.114 mg). However, the difference in whole-brain energy consumption (*P. occidentalis*: 1.76 µW; *P. rugosus*: 1.64 µW) is minimal owing to the much higher brain MSMR in the smaller-bodied species. Our study reveals that assessment of brain metabolism in a phylogenetic context can be a powerful tool to understand patterns of brain evolution across diverse animal clades by demonstrating the neurometabolic consequences of brain size variation.

## 4. Methods

### (a) Study species

Workers of 11 species (supplementary material, table S1) of *Pogonomyrmex* ants were collected from multiple colonies and locations in Arizona, California and Florida, USA, between May 2021 and January 2023. All species but *P. badius* are monomorphic. For the monomorphic species, workers were randomly sampled with no effort made to control for their sizes. For *P. badius*, which has minor/major workers, we sampled both sub-castes to ensure full coverage. Ants were housed in small plastic enclosures and stored in an environmental control room with the temperature fixed at 30°C. Ants were housed in groups of individuals from their original colony to prevent isolation stress. They were provided with 30% w/v sucrose solution, fresh water *ad libitum* and were processed for experimentation within three weeks of collection.

### (b) Sociometrics

We assessed the effect of social organization on brain mass and metabolism using colony size as an estimator of social complexity. Colony size is considered a reliable indicator of derived and advanced sociality in ants [52] and other eusocial insects [26,43,49,50] owing to its association with the potential number of interactions among workers and whether there is differential task specialization. Further evidence for colony size as a robust proxy of social complexity can be found in *P. barbatus*, where foraging dynamics are modulated by social interaction [96,97]. The members of these colonies experience high levels of social interaction and form some of the largest colonies in the genus.

### (c) Whole body resting metabolic rate measurements

To determine the whole-body MR of ants at rest, we used differential flow-through respirometry. The respirometer was in the same temperature-controlled (30°C) room the ants were stored in. We conducted all respirometry trials in the dark, with red filters applied to respirometry chambers to prevent incidental light from reaching the ants during preparatory lab work.

A Flowbar-8 mass flow meter system (Sable Systems, Las Vegas, NV, USA) pumped dry, CO_2_-free air from a gas cylinder into the respirometry chamber at a standard temperature and pressure flow rate of 50 ± 1 ml min^–1^. Ants were placed into a chamber consisting of a Bev-A-Line tube (length: 25 mm, diameter: 3.2 mm). Output from the chamber was directed to the sample cell of a LI-7000 CO_2_/H_2_O gas analyser, which sampled at 1 Hz and was calibrated with two CO_2_ calibration standards analysed ± 0.2 ppm.

We recorded a baseline measurement, without an ant, for at least one minute preceding each respirometry trial. After introducing the ant to the chamber, we covered the chamber with a transparent red filter and allowed the ant to adjust to the chamber until we observed little to no movement. We recorded CO_2_ production and synced this output with ant activity using a web camera (Logitech HD Pro Webcam C920, 1080 p) for 30 minutes. Individual ants were differentially active within the chamber, and the video recordings facilitated the selection of CO_2_ production during periods of inactivity to avoid any confounds owing to behaviour.

We digitized the analog output from the LI-7000 using a Sable Systems UI2 and recorded once per second using ExpeData (Sable Systems, v. 1.7.2) for Windows. We calculated average CO_2_ levels during time periods when we observed ants to be still.

CO_2_ production rates (ml h^–1^) were calculated as: VCO_2_ = FCO_2_ × FR, with FR equal to the flow rate (ml h^–1^), and FCO_2_ equal to the fractional CO_2_ level (µmol mol^–1^) in the excurrent air from the respirometry chamber. We did not measure the respiratory quotient of all our species. The respiratory quotient of worker ants has been reported as 0.71, 0.77, 0.80, 0.91, 0.92 and 1.02 [98–102]. We converted worker CO_2_ production to oxygen consumption using the average respiratory quotient of these studies (0.85) and then calculated MR in microwatts, assuming 20.4 joules ml oxygen^–1^ [103]. Ants were sacrificed by freezing after being weighed using an XPE56 XPE microanalytical balance (Mettler Toledo, Columbus, OH, USA). Brain and whole-body MRs were taken from separate individuals. Sample sizes for each species can be found in table S1 in the supplementary material.

### (d) Brain metabolic rate measurements

Brain oxygen consumption rates were measured *ex vivo* using a Seahorse XF HS Mini Analyser (Agilent, Santa Clara, California, USA) following methods outlined in [104]. Workers were anaesthetized on ice and their fresh masses recorded using an XPE56 XPE micro-analytical balance (Mettler Toledo, Columbus, OH, USA). While remaining unconscious from the cold, workers were decapitated. Brains were then removed intact and undamaged from the head capsule under a dissecting microscope in Seahorse XF base media (Agilent, Santa Clara, California, USA) supplemented with 0.01 mol l^–1^ glucose and sodium pyruvate. The Seahorse XF HS Mini Analyser has eight wells, two of which were left empty as control wells, leaving six that contained intact dissected brains. Assays were conducted at 32°C, as this was the temperature at which the Seahorse stabilized in the lab with the heater turned off and was very near the holding and respirometry temperature used.

Brain MRs were recorded in cycles. The Seahorse XF HS Mini mixes oxygen into the media within the well and then measures the rate at which oxygen is depleted, generating an oxygen consumption rate (picomols min^–1^). This cycle was repeated 12 times, taking approximately 1 hour; MRs were usually very stable over this period. The rates estimated from the last three cycles were selected and used to calculate a mean MR (supplementary material, figure S3). Samples that did not produce stable, linear oxygen consumption rates from the last three cycles were excluded from the analysis. Insect brains are believed to catabolize a mixture of carbohydrates and fatty acid metabolites [105]. In the absence of knowing the exact brain respiratory quotient, we converted oxygen consumption into µW using the same conversion factor we used for resting workers, 20.4 J ml^–1^ O_2_ consumed. After completion of the assay, brain mass was measured using an XPE56 XPE microanalytical balance (Mettler Toledo, Columbus, OH, USA). Brain and whole-body MRs were taken from separate individuals. Sample sizes for each species can be found in table S1 in the supplementary material.

### (e) Statistical analysis

All statistical analyses were conducted using R version 4.2.2 [106]. Phylogenetic signal was calculated using the package ‘phytools’ [107]; Brownian correlation structures for PGLS regressions were calculated using the package ‘ape’ [108] and data visualization was conducted using the package ‘ggplot2’ [109] and ‘ggtree’ [110]. Generalized additive models were implemented using the package ‘mgcv’ [111]; phylogenetic penalties were implemented using the package ‘MRFtools’ [112].

MRs and brain masses were analysed using linear models constructed using the R base package [106] or mixed-effects models constructed using the ‘lme4’ package [113]. Log_10_ body mass and log_10_ brain mass were modelled as fixed effects, with species modelled as a random intercept. Significance of terms in mixed-effects models was assessed using the package ‘lmerTest’ [114]. Where appropriate, MRs and body masses were log_10_ transformed to facilitate allometric analysis.

### (f) Phylogenetic reconstruction

Eleven samples from the 143-sample phylogeny inferred by Graber *et al.* [61] were used to construct the phylogeny used in our analyses. UCE library prep and enrichment protocols are described by Graber *et al.* [61]. A total of 851 alignments with >95% representation of the 11 taxa were concatenated into a single matrix using ‘phyluce_align_get_only_loci_with_min_taxa’. IQ- TREE2 was used to infer a maximum likelihood phylogeny with 1000 bootstrap replicates; the general time reversible and gamma distribution GTR+G model was used for the entire alignment, a flexible model that estimates six distinct substitution rates and takes rate heterogeneity into account while avoiding biases introduced by oversimplified models.

## Supplementary Material



## Data Availability

Data and code used in this manuscript have been deposited at OSF [115]. Supplementary material is available online [116].
