# Supplementary material for: Evolution of neurometabolic frugality in harvester ants
Source: Proc Biol Sci. 2026 Oct 21;293(2081):20260387. doi: 10.1098/rspb.2026.0387 (PMC13682931; doi:10.1098/rspb.2026.0387)
Supplement: Supplementary file 1 [file rspb.2026.0387.zip › rspb.2026.0387/rspb20260387_si_001.docx]

SUPPLEMENTARY MATERIAL

***Table S1****: Means, ranges and sample sizes of variables from workers of each species*

| **Species** | **Body mass (mg ± SD)** | **Brain mass (mg ± SD)** | **Body metabolic rate (μWatts ± SD)** | **Brain metabolic rate (μWatts± SD)** | **Colony size (workers per colony)** | **Colony size reference** |
| --- | --- | --- | --- | --- | --- | --- |
| *P. apache* | 12.35 ± 1.38  (n = 15) | 0.15 ± 0.02  (n = 11) | 16.09 ± 7.58 | 1.65 ± 0.08 | 80 | Cole (1954) [1] |
| *P. badius* | 14.12 ± 9.48  (n = 19) | 0.14 ± 0.03  (n = 19) | 11.52 ± 9.41 | 1.72 ± 0.11 | 5000 | Tschinkel (2017) [2] |
| *P. barbatus* | 14.95 ± 2.87  (n = 51) | 0.19 ± 0.01  (n = 17) | 19.74 ± 12.27 | 1.68 ± 0.05 | 12000 | Gordon (1992) [3] |
| *P. californicus* | 6.08 ± 0.78  (n = 13) | 0.11 ± 0.06  (n = 15) | 6.75 ± 4.35 | 1.60 ± 0.13 | 3250 | RA Johnson, pers. obvs |
| *P. desertorum* | 6.03 ± 0.41  (n = 36) | 0.10 ± 0.01  (n = 15) | 5.66 ± 2.45 | 1.64 ± 0.15 | 500 | Creighton (1956) [4] |
| *P. huachucanus* | 4.29 ± 2.63  (n = 26) | 0.08 ± 0.01  (n = 7) | 4.11 ± 2.23 | 1.60 ± 0.1 | 150 | Creighton (1952) [5]; RA Johnson, pers. obs. |
| *P. imberbiculus* | 2.12 ± 0.24  (n = 12) | 0.06 ± 0.02  (n = 13) | 4.91± 1.56 | 1.45 ± 0.07 | 125 | Heinze, et al. (1992) [6] |
| *P. maricopa* | 9.80 ± 1.75  (n = 16) | 0.12 ± 0.02  (n = 10) | 5.86 ± 1.31 | 1.63 ± 0.08 | 750 | RA Johnson, pers. obs. |
| *P. occidentalis* | 7.91 ± 1.34  (n = 14) | 0.11 ± 0.03  (n = 17) | 9.20 ± 5.29 | 1.76 ± 0.1 | 3880 | Lavigne (1969) [7] |
| *P. pima* | 1.68 ± 0.29  (n = 36) | 0.05 ± 0.01  (n = 5) | 5.02 ± 4.24 | 1.41 ± 0.08 | 250 | Johnson, et. al (2007) [8] |
| *P. rugosus* | 15.87 ± 6.65  (n = 14) | 0.29 ± 0.03  (n =7) | 29.81 ± 33.96 | 1.64 ± 0.04 | 8600 | MacKay (1981) [9] |

1.	Cole AC. 1954 Studies of New Mexico ants. IX. *Pogonomyrmex apache* (Wheeler) a synonym of *Pogonomyrmex sancti-hyacinthi* (Wheeler) (Hymenoptera: Formicidae). *Journal of the Tennessee Academy of Sciences* **29**, 266–271.

2.	Tschinkel WR. 2017 Lifespan, age, size-specific mortality and dispersion of colonies of the Florida harvester ant, *Pogonomyrmex badius*. *Insectes Soc.* **64**, 285–296. (doi:10.1007/s00040-017-0544-0)

3.	Gordon DM. 1992 Nest relocation in harvester ants. *Ann. Entomol. Soc. Am.* **85**, 44–47.

4.	Creighton WS. 1956 Studies on the North American representatives of *Ephebomyrmex* (Hymenoptera: Formicidae). *Psyche (Camb. Mass).* **63**, 54–66. (doi:10.1155/1956/90103)

5.	Creighton WS. 1952 Studies on Arizona ants (3) the habits of *Pogonomyrmex huachucanus* (Wheeler) and a description of the sexual castes. *Psyche (Camb. Mass).* **59**, 71–81. (doi:10.1155/1952/64756)

6.	Heinze J, Hölldobler B, Cover SP. 1992 Queen polymorphism in the North American harvester ant, *Ephebomyrmex imberbiculus*. *Insectes Soc.* **39**, 267–273. (doi:10.1007/BF01323947)

7.	Lavigne RJ. 1969 Bionomics and nest structure of *Pogonomyrmex occidentalis* (Hymenoptera: Formicidae). *Ann. Entomol. Soc. Am.* **62**, 1166–1175. (doi:10.1093/aesa/62.5.1166)

8.	Johnson RA, Holbrook CT, Strehl C, Gadau J. 2007 Population and colony structure and morphometrics in the queen dimorphic harvester ant, *Pogonomyrmex pima*. *Insectes Soc.* **54**, 77–86. (doi:10.1007/s00040-007-0916-y)

9.	MacKay WP. 1985 A comparison of the energy budgets of three species of *Pogonomyrmex* harvester ants (Hymenoptera: Formicidae). *Oecologia* **66**, 484–494. (doi:10.1007/BF00379338)

***Figure S1:*** *Worker masses increase with increasing social complexity. Mean worker mass increases with increasing colony size (worker mass = 0.000000634 * colony population + 0.004862386).*

**Fig. S2: Body mass corrected metabolic rates as a response to colony size. A)** Body mass-corrected whole-body metabolic rate (residuals of whole-body MR regressed against body mass, Fig. 4A) as a response to mean colony population. **B)** body-mass-corrected brain MSMR (residuals of brain MSMR regressed against body mass, Fig. 5D) as a response to mean colony population.

**Fig. S3:** Representative brain metabolic rate data from Agilent Seahorse. The rates estimated from the last three cycles were selected and used to calculate a mean MR (Fig. S1). Samples which did not produce stable, non-linear oxygen consumption rates from the last 3 cycles were excluded from the analysis.
